# The Molecular Mechanisms of Gametic Incompatibility in Invertebrates

**DOI:** 10.32607/20758251-2019-11-3-4-15

**Published:** 2019

**Authors:** A. A. Lobov, A. L. Maltseva, N. A. Mikhailova, A. I. Granovitch

**Affiliations:** Department of Invertebrate Zoology, Faculty of Biology, St Petersburg State University, Universitetskaya Emb. 7/9, St. Petersburg, 199034, Russia; Laboratory of Regenerative Biomedicine, Institute of Cytology of the Russian Academy of Sciences, Tikhoretsky Ave. 4, St. Petersburg, 194064, Russia; Centre of Cell Technologies, Institute of Cytology of the Russian Academy of Sciences, Tikhoretsky Ave. 4, St. Petersburg, 194064, Russia

**Keywords:** gamete recognition proteins, gametic incompatibility, gametic isolation, reproductive isolation, speciation, invertebrates

## Abstract

Fertilization (gamete fusion followed by zygote formation) is a multistage process. Each stage is mediated by ligand-receptor recognition of gamete interaction molecules. This recognition includes the movement of sperm in the gradient of egg chemoattractants, destruction of the egg envelope by acrosomal proteins, etc. Gametic incompatibility is one of the mechanisms of reproductive isolation. It is based on species-specific molecular interactions that prevent heterospecific fertilization. Although gametic incompatibility may occur in any sexually reproducing organism, it has been studied only in a few model species. Gamete interactions in different taxa involve generally similar processes, but they often employ non-homologous molecules. Gamete recognition proteins evolve rapidly, like immunity proteins, and include many taxon-specific families. In fact, recently appeared proteins particularly contribute to reproductive isolation via gametic incompatibility. Thus, we can assume a multiple, independent origin of this type of reproductive isolation throughout animal evolution. Gametic incompatibility can be achieved at any fertilization stage and entails different consequences at different taxonomic levels and ranges, from complete incompatibility between closely related species to partial incompatibility between distantly related taxa.

## INTRODUCTION

The modern interpretation of species identity is based on the idea of unity of the species gene pool [1-3]. The hypotheses describing the mechanisms of speciation (microevolution) refer to the potential mechanisms of species subdivision into either partially or completely reproductively isolated groups [3]. Reproductive isolation (RI) is an essential stage of speciation and, at the same time, the key species criterion [1-3].

RI is realized via prezygotic and postzygotic mechanisms that are triggered at the stages that precede and follow zygote formation, respectively [3]. Their biological roles differ: the prezygotic and reproductive barriers form and function at early stages of speciation; postzygotic – at the late stages [4-7]. For example, it took at least 22 million years of divergence for the postzygotic RI between closely related bird species to form [3]. On the contrary, the prezygotic reproductive barriers between the Drosophila species can form within less than ten generations [8]. Gametic incompatibility (GI) is one of the prezygotic reproductive barriers that might emerge rather quickly [3].

GI is based on interactions between highly specialized gamete-recognition molecules. Gamete recognition proteins (GRPs) are expressed in reproductive tissues and are typically uninvolved in other functions [9, 10].

Even single amino acid substitutions in GRPs influence the efficiency and/or species specificity of gamete recognition [9, 10]. For example, it is considered that as few as 10 amino acid changes in the sea urchins acrosomal protein bindin can lead to RI between two species [11]. Remarkably, the GRP structure is modified by some forms of natural selection, leading to an adaptive high level of GRP polymorphism; along with the immunity proteins, GRPs are among the most rapidly evolving traits [9-20].

Investigation of the individual mechanisms of RI at the molecular level has now become possible: the postgenomic era offers novel tools for studying the genomes and proteomes of many organisms. However, many of the proteins involved in RI belong to novel families and their secondary structure and/or functions cannot be adequately predicted using the available bioinformatic resources.

Sea urchins and marine mollusks (genus Haliotis) are the model objects routinely used for GI studies in externally fertilizing species [9, 10]. In some other invertebrates, only distinct stages of gamete recognition were studied (this will be discussed below). In many high-level invertebrate taxa, no molecular mediators of gamete recognition have been detected yet. For example, the recently described paraspermal protein LOSP became the first identified potential GRP in caenogastropods [21, 22].

## GAMETE RECOGNITION

**Fig. 1:**
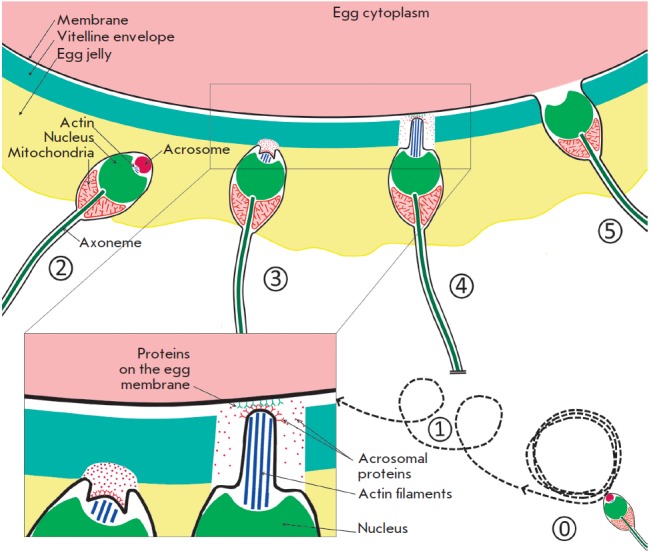
Stages of gamete recognition in sea urchins. Numbers denote the steps of gamete recognition, with the description provided in the main text

Gametic incompatibility is based on the structural changes that take place in GRM to ensure their specific interaction. The principal mechanisms of gamete recognition are similar in different organisms (with a few exceptions, such as Nematoda) and involve five stages (Fig. 1, exemplified by the sea urchin) [9, 23].

**Stage 1: sperm guidance (steps 0–1)**

**Fig. 2:**
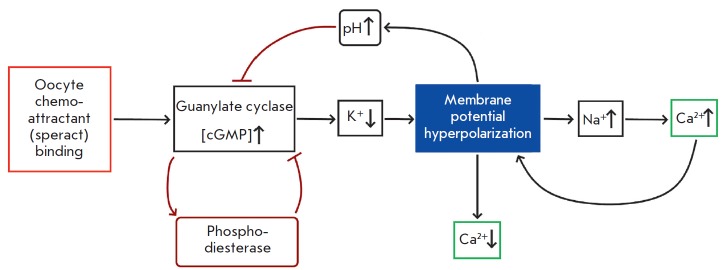
Schematic illustration of the signaling cascade that is activated by speract, a sea urchin egg chemoattractant. The illustration was adapted from [28]

At the start of this stage, after a period of spontaneous movement of a spermatozoon (usually following a wide loop without linear sections) (Fig. 1, step 0), a sperm guidance program is initiated (Fig. 1, step 1; [24]). The action of egg chemoattractants forces the sperm to move linearly with sharp loop-shaped turns [24]. This sperm movement pattern has been demonstrated for many phylogenetically distant taxa, such as echinoderms, chitons [25], cnidaria [25, 26], and polychaete Arenicola marina [27]. It is made possible by molecular mechanisms similar to those in the sea urchins Strongylocentrotus purpuratus (Fig. 2) [28].

In sea urchins, the egg chemoattractant (speract) activates the guanylate cyclase receptor on the sperm membrane, resulting in the emergence of cGMP opening of the cGMP-dependent K+ channels (Sp-tetraKCNG). Opening of these channels causes membrane hyperpolarization and activates the signaling cascade that drives calcium concentration oscillations [28]. The oscillating pattern of the signal results in an alternation of the individual phases of sperm movement: the spermatozoon moves linearly at low Ca^2+^ concentrations while assuming a sharp loop-shaped turn at high Ca^2+^ concentrations [29].

**Table 1:** A list of egg chemoattractants detected in invertebrates and protists

Taxon	Species	Chemoattractant	Reference
Cnidaria	Montipora digitata; Lobophytum crassum	Unsaturated fatty alcohols; macrocyclic diterpene alcohols	[30, 31]
Echinodermata		Peptides	[32, 33]
Mollusca	Octopus vulgaris; Sepia officinalis		[34, 35]
	Haliotis	L-tryptophan	[36]
Ascidia	Ciona intestinalis	Sulfated steroids	[37]
Nematoda	Caenorhabditis elegans	Polyunsaturated fatty alcohols (PUFAs)	[38]
Brown algae	Fucus vesiculosus	Unsaturated carbohydrates (fucoserratins)	[39, 40]
Infusoria	Euplotes	Proteins	[41]

The structure of egg chemoattractants is unique in all the studied taxa (Table) [30-41]. It is possible that different sperm guidance systems form independently based on the fundamental mechanism of sperm motility [42].

**Stage 2–3: the acrosome reaction and destruction of the egg envelope (steps 2–4)**

The key stage of fertilization is the penetration of a spermatozoon through the egg envelope ensured by acrosomal proteins. These specialized proteins reside in the acrosome, a vesicle in the apex of the sperm [43]. In most animals, spermatozoa have a relatively large acrosome; however, the acrosome can also be rather small (e.g., in filiform spermatozoa of Littorina mollusks and Lepisma insects) [44, 45].

Acrosomal proteins are released upon acrosomal exocytosis during the acrosome reaction (AR; step 2). Also in many animals, such as sea urchins, pH-dependent actin polymerization occurs and an acrosomal rod forms (Fig. 1, 3-4) [46, 47]. AR is triggered by the interaction between specific sperm receptors and their ligands in the egg envelope.

Among invertebrates, the molecular basis of these processes has been studied only in echinoderms. Yet, they probably differ among invertebrate taxa. For example, these processes vary even within deuterostomes. In sea urchins and mammalians, AR is induced by different classes of molecules: sulfated polysaccharides and glycoproteins ZP3, respectively [46, 48]. As a result, non-homologous proteins are responsible for the recognition of these compounds: the 210 kDa membrane glycoprotein (REJ) acts as a receptor in sea urchins; PKDREJ and β-galactosyl transferase act as receptors in mice [46, 48].

In humans, the acrosome reaction can be induced not only by glycoprotein ZP3, but also by ZP1 and ZP4; additional receptors also seem to be involved [49]. Unlike the activation of AR via the ZP3 pathway, activation via the ZP1 and ZP4 does not involve G-protein signaling cascades and activates the L- and T-type voltage-gated calcium channels [49]. Hence, the signaling cascades inducing AR significantly differ within mammals; furthermore, they seem to use several independent pathways for their activation.

**Fig. 3:**
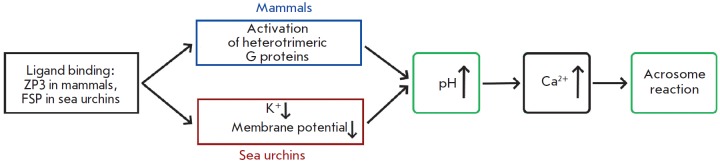
Schematic illustration of the signaling cascades inducing the acrosome reaction in sea urchins and mammals. The illustration was adapted from [48]

However, there also are fundamental similarities between the signaling cascades that induce AR in mammals and sea urchins (Fig. 3) [48]. Although their receptors belong to different classes, they induce the opening of calcium channels and those that cause a local increase in pH. These factors activate phospholipase C. The emergence of IP3 causes the release of intracellular calcium, the opening of the calcium channels controlled by the Ca^2+^ release (SOCs), and induction of AR.

Further, the course of fertilization depends on the activity of acrosomal proteins. In the organisms studied so far, these proteins are non-homologous but can be clearly subdivided into three functional groups.

(1) Components that degrade the egg envelope. These components usually are proteases or other enzymes [9, 10,50], but the integrity of the egg envelope can be lost even without the rupture of covalent bonds.

The acrosome of abalones (genus Haliotis) contains the 16 kDa lysin protein that degrades the egg envelope via the non-enzymatic mechanism. Lysin contains five α-helices that form two surfaces: a hydrophobic surface on one side and a cationic surface on the other. Lysin exists in the form of dimers noncovalently bound due to hydrophobic surfaces. The cationic surface sticks outside the dimer and is responsible for the interaction with VERL.

The vitelline envelope of abalones eggs consists of dense fibers that contain 6–10 VERL glycoprotein molecules, each; its structure is stabilized by hydrogen bonds. The interaction between the lysin dimer and VERL repeats causes lysin monomerization and binding to VERL. This specific recognition replaces the hydrogen bonds between VERL molecules with VERLlysin hydrogen bonds, causing local degradation of the egg envelope [51-53].

(2) Components that ensure sperm adhesion to the egg envelope. Bindin discovered in sea urchin sperm was the first protein known to have this function [13]. In different sea urchin species, the size of mature bindin ranges from 193 to 418 amino acids. It consists of a 55-amino-acid-conserved core, which is involved in gamete fusion (stage 4), and two flanking regions responsible for species-specific adhesion to the egg envelope [13]. Non-homologous proteins with a similar function were detected in the echiuran (spoon worm) Urechis sp. [54]. Five highly homologous lectins serve this function in oysters [55].

(3) Components that affect the cell physiology. For example, the acrosomal proteins M3, M6, and M7 in bivalve mollusks of the genus Mytilus induce completion of oocyte meiosis [56] while bindin in spoon worm activates the oocyte [54].

**Stage 4: membrane fusion (step 5)**

After local degradation of the outer envelope of the egg, the membranes of interacting gametes approach each other and fuse. The lipid composition of these membranes, especially the cholesterol concentration [57], may affect the fusion process [58]. However, the key role is played by specialized proteins. It is assumed that HAP2, a homologue of the class II viral fusion protein, is involved in gamete fusion in eukaryotes [59]. It was demonstrated experimentally that this protein participates in gamete fusion in sea anemones Nematostella vectensis [60], in angiosperms belonging to the genus Arabidopsis [61], and protists Chlamydomonas, Tetrahymena, and Plasmodium [62, 63]; the orthologous genes of protein HAP2 were detected in the genomes of almost all metazoans [64]. In addition to HAP2, there are data on the involvement of group-specific proteins in membrane fusion (e.g., bindin from sea urchins, which has already been mentioned) [65-67].

Gamete recognition is based on conserved processes controlled by second messengers (primarily by calcium ions). However, a large number of non-homologous proteins are involved in gamete recognition in distantly related taxa. Its complexity has increased multiple times throughout the evolution of individual taxa.

## THE MECHANISMS OF GAMETE INCOMPATIBILITY IN EXTERNALLY FERTILIZING INVERTEBRATES

GI is studied in detail in a model of closely related sea urchin species. For other taxa, data exists only for individual stages; unfortunately, the reasons why GI evolves at specific stages of gamete recognition have been elucidated in none of the models.

**Peptide chemoattractants**

(Fig. 1, steps 0–1) in sea urchins often display species-specific differences in their amino acid sequences [68]. The species specificity of sperm guidance was confirmed by experimental data collected on 17 species from several sea urchin genera [69]. For example, the chemoattractant of Arbacia punctulata has no effect on S. purpuratus or Lytechinu spictus sperm [70-72].

A similar phenomenon has been observed in several holothurian species belonging to the Bohadschia genus and 22 ophiuroid species [73]. However, there are a number of examples when chemoattractants exhibit no species-specific activity. In a number of holothurian species (e.g., Cucumaria piperata), spermatozoa respond not only to the egg chemoattractants of closely related species, but also to starfish eggs [74, 75]. In echinoderms, the specificity of sperm guidance varies from the species level to the absence of specificity within the class. The reason for these observed differences remains unknown.

Total extracts of the reproductive tissues of the bivalve mollusks Dreissena polymorpha and D. bugensis can guide both homo- and heterospecific sperm, but the chemoattractant concentration has to be 100-fold higher in order to guide heterospecific gametes [75]. A similar situation is typical for sea anemones of the genus Montipora: experiments with three synthetic analogues of chemoattractants demonstrated that the spermatozoa of different species vary in their response to different concentrations of these substances [30]. Finally, it has also been reported that, sometimes, the chemical structure of egg chemoattractant can be identical in the groups that are being studied, since it is involved in basic physiological processes. For instance, this is true for L-tryptophan (a chemoattractant in Haliotis), which is considered to release in its intact form [36].

**Induction of AR**

(Fig. 1, steps 2–3) in sea urchins may also be species-specific. This is made possible by the differences in the position and number of sulfate groups in the polysaccharide chains of the sulfated polysaccharides of the egg envelope [15].

In starfish, specificity of AR induction exists only at the subfamily level (e.g., between the species belonging to the genera Asterias and Aphelasterias (Asteriinae subfamily)) [76]. Furthermore, in most species AR can be induced by many nonspecific interactions, such as mechanical contact with a microscope slide.

**Enzymatic degradation of the egg envelope**

(Fig. 1, step 4) in sea urchins appears to be not species-specific [77]. It is believed that in most cases of heterospecific fertilization between closely related species, sperm adhesion to the egg envelope is disrupted [78-82]. For example, in experiments on heterospecific gamete interactions among 11 sea urchin species AR induction occurred in nine combinations, but heterospecific adhesion was observed in none of the cases [80, 81]. It has been confirmed that the variability in bindin plays a crucial role in the species specificity of these processes in sea urchin species that dwell in the same habitat (genera Echinometra, Heliocidaris, and Strongylocentrotus) [83-85]. Species specificity is achieved through structural matching between the flanking regions of bindin and its receptor, EBR1. Alleles characterized by different interaction efficiencies have been revealed in the sea urchin population; however, a model that interprets the matching between them has not yet been developed.

**Fig. 4:**
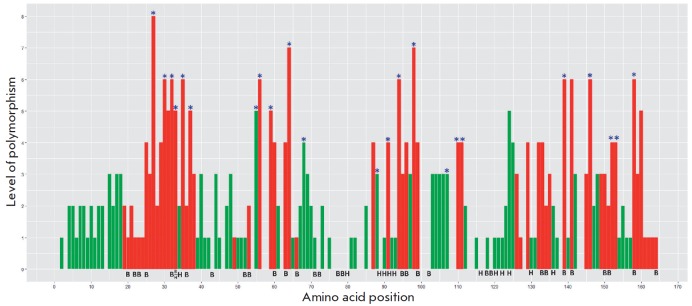
Diagram showing the variability of the primary structure of the lysin protein. The analysis included the lysin sequences of 25 species from two families (Trochidae and Haliotidae, [53]). The amino acid position in the molecule is plotted against the X axis; the number of detected substitutions is plotted along the Y axis. The sites of radical substitutions (replacement of a hydrophobic amino acid with a hydrophilic one, a cationic amino acid with an anionic one, or deletions) are marked in red. * – sites influenced by positive selection. H – hydrophobic amino acids forming a hydrophobic surface; B – basic amino acids that interact with VERL

Species-specific egg-envelope degradation has been revealed only in abalones (Haliotis spp.), but not in other animal taxa. As mentioned above, acrosomal protein lysin degrades the egg envelope via specific interactions with VERL. The lysin protein either does not dissolve the envelope of heterospecific eggs at all or is inefficient in in vitro experiments with the eggs of three abalone species (H. rufescens, H. cracherodii, and H. corrugata) [86]. This specificity is due to the respec tive mutations in VERL repeats and in the positively charged lysin region that carries 24 cationic amino acid residues, of which only seven are conserved (Fig. 4).

Finally, there are data showing species-specific differences in the acrosomal proteins in oysters and mussels: it has been hypothesized that the polymorphism in acrosomal proteins maintains the reproductive barriers that exist between the closely related species Crassostrea [54, 87] and Mytilus [88].

The species specificity of ***membrane fusion*** (Fig. 1, step 5) has been reported (e.g., upon heterospecific fertilization of gametes in the sea urchins Echinometra mathaei and E. oblonga [78]). However, this species specificity appears to be related to some unknown factors, rather than to the structural differences in protein HAP2, which controls gamete fusion in all eukaryotes [58, 59].

Therefore, the variability of individual gamete recognition proteins can reduce fertilization efficiency and even cause GI. GI can occur at any stage of gamete interaction.

In invertebrates, GI is implemented at different taxonomic levels. In a number of studied taxa, it is inefficient at the species level [30, 68, 69, 73-75]. Unequivocal examples of GI between closely related species have been reported only for complexes of closely related species: in the mollusk genera Haliotis and Tegula and the sea urchin genera Echinometra, Heliocidaris, and Strongylocentrotus. Heterospecific fertilization is often possible but is not as efficient as the interactions between homospecific gametes; in particular, this was demonstrated in no-choice experiments [52, 75, 77, 89-91]. It is possible that in a number of the studied taxa, GI between closely related species is achieved only upon competition between homo- and heterospecific gametes.

It is expected that genome-wide sequencing and whole-genome annotation of many species listed in this section, as well as advances in bioinformatic methods for predicting the secondary structures of sought-after proteins, will lead to some breakthrough in this field.

## GAMETIC INCOMPATIBILITY IN INTERNALLY FERTILIZING SPECIES

In internally fertilizing species, gamete interaction largely depends on the physiological state of a female. For example, oocyte maturation in insects is induced by the synthesis of vitellogenin, whose secretion is regulated by a juvenile hormone, ecdysosteroid, and a number of nutritional signals [92]. The female im mune status also plays an important role as it affects sperm storage and survival. The more active the female immune system is, the shorter the sperm storage period will be (see review [93]). This phenomenon is clearly visible in insects that mate only once in their life. A high activity of the immune system of an Atta colombica ant queen has a negative effect on sperm survival; in order to ensure long-term sperm storage, special mechanisms that suppress immunity are activated in the female’s organism [94]. To gauge the activity of the immune system, Baer et al. measured the efficiency of encapsulation response: small pieces of nylon were inserted into a female’s body at equal time periods, and the number of melanized haemocytes encapsulating this extraneous object was counted [94]. This method provides only indirect evidence to the correlation between insects’ immune systems and sperm storage, and it still remains to be elucidated what specific molecular cascades are involved in these processes.

In internally fertilizing animals, it would be more accurate to use the term “post-copulatory pre-zygotic reproductive barriers (PCPZ)” instead of GI. This concept involves a number of mechanisms of reproductive isolation that have similar manifestations but are based on different molecular cascades.

PCPZ is often based on a male ability to affect a female physiology. For example, in Anastrepha suspensa flies, male presence increases the rate of female ovarian development [95]. Another example is the phenomenon of nuptial gift transfer to females, which affects their physiology and the mating rate (see review [96]). Seminal fluid proteins transferred by the male during internal fertilization play an important role. For instance, the seminal fluid components of the moth Heliothis virescens stimulate the female to produce oocytes [97]; the specific protein inducing oocyte production in homospecific females is also known in the cricket Allonemobius [98, 99]. We believe that, despite their functional similarity, these proteins are non-homologous.

It has been demonstrated that seminal fluid proteins are rather diverse in terms of their functions and structure. For example, at least 127 proteins were found in the seminal fluid of the beetle Callosobruchus maculatus [100]. Seminal fluid proteins may affect oocyte production and changes in the shape of reproductive ducts; they also ensure antimicrobial activity and female receptivity. The proteins can determine the period of sperm storage and modulate the activity of spermatozoa, thus influencing potential sperm competition (SC). Finally, these proteins were shown to be involved in the blocking of the spermatheca (via the formation of mating plugs, see review [101]). A proproteomic analysis of seminal fluid components is quite relevant, since many seminal fluid proteins belong to novel families with unknown functions. The available data do not allow one to perform a comparative structural analysis and thoroughly evaluate their role in reproduction. For example, 19 previously not-annotated proteins with unknown functions were discovered in 2009 by proteomic analysis of the seminal fluid of the fruit fly (one of the most commonly used model organisms), followed by a bioinformatic analysis of the whole-genome data [102]. This problem is likely to find a solution as bioinformatic algorithms for predicting protein structure and function based on their primary structure are developed.

Nonetheless, the PCPZ strategy involves the same principles of ligand–receptor interactions. Like in gamete incompatibility, PCPZ species specificity is caused by the coevolution of individual pairs of molecules. These mechanisms may be classified into two groups: female cryptic choice and sperm competition.

**Sperm competition (SC)**

Polyandry (multi paternity) is a phenomenon that materializes when spermatozoa from several homospecific or sometimes heterospecific partners enter a female reproductive system. Seminal fluid is involved in the formation/sustaining of the active state of the spermatozoa; its components can determine the probability of oocyte fertilization. When sperm from heterospecific males comes into contact, seminal fluid components may be responsible for the outcome of sperm competition (see reviews [103, 104]). This competition may result in conspecific sperm precedence. For example, single mating between individuals from closely related Drosophila species may result in interspecific hybridization. However, when a female mates with a hetero- and a homospecific males, most of the progeny will come from the homospecific male [105]. It has been demonstrated experimentally that this effect is connected with seminal fluid proteins [105]. These mechanisms have been extensively studied in a pair of closely related species: Drosophila simulans and D. mauritiana. PCPZ between them is based on two mechanisms that depend on the copulation order. (1) If homospecific copulation is the first to occur, the seminal fluid components inactivate the heterospecific sperm subsequently entering the female reproductive system. (2) If heterospecific copulation is the first to occur, subsequent homospecific mating results in physical displacement of heterospecific sperm from the sperm storage organs [106]. A similar phenomenon has been demonstrated in flour beetles [107], crickets [108], beetles Callosobruchus [109], dragonflies [110], and ladybugs [111].

**Female cryptic choice**

Female cryptic choice (FCC) is a combination of behavioral, anatomical, and physiological features that allow a female to control efficiency in the process of reproductive products transferring (precopulative FCC) or fertilization (postcopulative FCC; see review [112]). For example, upon the mating of the yellow dung flies Scathophaga stercoraria, the probability of egg fertilization depends on which spermatheca the sperm has entered. The female controls sperm distribution, thus rendering the contributions of males to the progeny unequal [113]. Furthermore, the components of female accessory reproductive glands affect sperm survival, which varies in males with different genotypes [113].

Identically to SC, FCC is responsible for conspecific sperm precedence [112]. For example, after the mating of crickets belonging to two species Allonemobius fasciatus and A. socius, heterospecific sperm loses its motility in the female reproductive system [108].

It is clear that the SC and FCC strategies are phenomenologically similar: so, it is challenging to pinpoint the specific mechanisms that are responsible for reproductive isolation. For example, a female of L. saxatilis belonging to the currently being studied group of promiscuous, closely related species of the genus Littorina stores sperm and can simultaneously carry progeny from 20 or even more males. However, the distribution of embryonic genotypes in that case is quite abnormal: most of the progeny would originate from one to several males [114]. This phenomenon is considered to result from SC [114]. We presume that it could be related to the recently revealed paraspermal (i.e., residing in “paraspermatozoa”, type of sperm cells that are incapable of fertilization but are present in the sperm) protein LOSP and seminal fluid proteins [21, 22]. However, we possess no direct evidence of this yet.

## THE EVOLUTIONARY INTERPRETATION OF GAMETIC INCOMPATIBILITY

GRP polymorphism limits panmixia (random mating) in populations of externally and internally fertilizing invertebrates. Thus, coevolution of individual protein pairs has a direct effect on speciation. The phenomenon of rapid GRP evolution, which has been widely discussed in reviews published since 2002, warrants special attention [9, 10, 12]. The observed level of GRP polymorphism in a number of organisms, ranging from protists to metazoans, is much higher than the expected. Nonsynonymous substitutions in the genes encoding these proteins within the population occur more often than the synonymous ones (dN/dS > 1). This means that selection acts on the analyzed loci and the level of polymorphism in the respective proteins is potentially high [9, 10, 115]. For instance, this is true for pheromones of Euplotes and Basidiomycota, acrosomal proteins lysin in mollusks Tegula and Haliotida, and bindin in sea urchins [9, 10, 12]. The evolutionary significance of this phenomenon may be interpreted from two perspectives: explaining the reasons for the high level of GRP polymorphism and analyzing the role of GRP polymorphism and GI in speciation.

**The reasons for the high level of GRP polymorphism**

Although modifications in GRPs significantly reduce fertilization efficiency, there probably are some factors that form/maintain a high level of polymorphism.

*Sympatry* (coexistence of species in habitats overlapping either completely or partially) is tightly associated with a rapid rate of GRP evolution. Thus, GI and dN/ dS GRP > 1 are observed only between the sympatric sea urchin species of the genera Echinometra, Heliocidaris, and Strongylocentrotus [11, 13, 14, 15]. A similar situation is also typical of most of the other taxa mentioned in this article in which GI was demonstrated at the species level. In insects, the condition for GI is not just sympatry, but also polygamy (a reproductive strategy when a female can mate with several males, sometimes as many as a few dozen).

*Reinforcement* is a special form of selection driving reproductive isolation between spatially subdivided subpopulations within one species, which are adapted to different microniches. We have found only one published experimental confirmation that GI and reinforcement are linked. Under conditions of experimental sympatry in D. yakuba and D. santomea from allopatric populations (sympatric populations are also known for these species), ethological isolation and PCPZ become significantly stronger within four generations [8].

The discovered polymorphism in protein LOSP, which is potentially involved in RI in closely related sympatric species of the genus Littorina, probably serves as additional evidence of a connection between GI and reinforcement [21, 22]. According to our preliminary data, LOSP polymorphism is maximal in populations of L. *saxatilis*. This species exhibits a strong potential to forming races and local ecotypes [115-119] and exists sympatrically with the genetically closely related cryptic species L. *arcana* and L. *compressa* [120-122]. By contrast, this protein is virtually monomorphic in L. *obtusata* populations that exist sympatrically with L. fabalis but form no ecotypes in the analyzed populations.

One can assume that the high likelihood of a contact between heterospecific gametes or hybridogenesis between closely related subspecific groups makes the high level of GRP polymorphism and GI formation adaptive [8, 122-126].

*Interspecific sexual conflict* can also increase the level of GRP polymorphism in a population [18, 19, 127-129]. This model is based on simple stochastic principles: the likelihood of fertilization of the passive partner (the egg) by a spermatozoon is always high, while the spermatozoa compete for the fertilization of a specific egg. For an egg, the highest risk is polyspermia: so, it is adaptive to reduce the efficiency of gamete interaction. For a spermatozoon, the highest risk is competition with other spermatozoa: so, it is adaptive to an increase in the efficiency of gamete interaction. This conflict may result in high GRP polymorphism in the population [18, 19, 127-129], and it may also imply a molecular arms race between a spermatozoon and an egg.

**Relationship between the GRP polymorphism and speciation**

Taking into account the aforedescribed effect of single amino acid substitutions in GRP for GI, the high level of GRP polymorphism maintained in a population will inevitably partially limit random mating (panmixia).

*Speciation is primary.* The assumption that selection against hybrids directly influences the GRP polymorphism correlates well with one of the first definitions given for this form of selection by E. Mayr (1970): formation of reproductive isolation between two taxa would be adaptive if the hybrids are less well adapted than their parents [124]. The GRP genes are among the few loci whose products are either predominantly or exclusively related to fertilization: this very part of the genome can be the most “sensitive” to selection against hybrids [8, 125-127]. This point of view significantly contributes to our conventional model of ecological speciation. The phenomenon of high GRP polymorphism as a direct result of selection against hybrids explains the mechanisms of formation of reproductively isolated taxa and confirms the mere possibility of ecological speciation in sympatric populations.

*Limitation of panmixia is primary.* The data on the potential association between the GRP polymorphism and speciation can also be interpreted in the opposite direction. The subdivision of gene pools may be caused by “background” processes that are not directly involved in speciation. Sexual conflict can be such a factor. In this case, the intraspecific competition will form the primary genetic subdivision. This interpretation is supported by the fact that formation of intra- and interspecific SC in fruit flies is accompanied by similar genomic changes in the same loci [130].

Although the opinions presented in this review seem contradictory, they are in fact largely complementary. On the one hand, sexual conflict reduces the stability of the gene pool of species due to a high level of GRP polymorphism. On the other hand, selection against hybrids may lead to a “targeted” formation of reproductively isolated groups. According to the ecological speciation concept, any form of RI is adaptive and GI in particular arises given certain prerequisites – biological characteristics of individual taxa, such as polyandry.

Verification of these concepts is quite challenging and requires the development of novel model systems. The closely related, internally fertilizing species of marine mollusks from the genus Littorina (Mollusca: Caenogastropoda) may serve as such a model. This group has been comprehensively studied in respect to ecological speciation, local adaptation, reproductive behavior, parasite–host interactions, etc. [116-122, 131-133]. Potential effectors of gamete recognition (e.g. paraspermal protein LOSP involved in RI between closely related species via one of the mechanisms described above, such as SC) are currently being actively researched [21, 22, 118]. At least several dozen novel seminal fluid proteins, potentially involved in the formation of interspecific reproductive barriers, have already been discovered, and we plan to report on them in the near future.

## CONCLUSIONS

In all studied species, gamete recognition goes through the same stages; however, the stages are based on non-homologous proteins in phylogenetically distant taxa. GI can emerge at any step of gamete recognition, due to structural changes in the respective molecules, and can be observed at various taxonomic levels: between members of different classes, at the genus level, between closely related species, and even at the intraspecific level.

Despite the wide use of whole-genome sequencing, studying novel, highly variable protein families is a challenging task; therefore, the data on GRPs is still fragmentary.

The key trends in this field are related to (1) developing new model systems belonging to different taxonomic groups and manually annotating novel protein families and (2) improving our bioinformatics algorithms for automated annotation and prediction of protein structure and function.

## Abbreviations

GRM/P: gamete recognition molecules/proteins
GI: gametic isolation
AR: acrosome reaction
FCC: female cryptic choice
SC: sperm competition
RI: reproductive isolation
PCPZ: post-copulatory prezygotic reproductive barriers
